# Mode of Mechanical Ventilation in a Case of Venolymphatic Malformation: Spontaneous-Saves, Positive-Precludes

**DOI:** 10.4274/TJAR.2023.221115

**Published:** 2023-08-18

**Authors:** Prateek Arora, Subrata Kumar Singha, Omer Md Mujahid, Snigdha Kumari, Abinaya Prakashbabu

**Affiliations:** 1 Department of Anaesthesiology, Pain and Critical Care, All India Institute of Medical Sciences (AIIMS), Raipur, India; 2 Department of Anaesthesia & Critical Care, All India Institute of Medical Sciences (AIIMS), Raipur, India; 3 Department of Anaesthesiology, All India Institute of Medical Sciences (AIIMS), Raipur, India

**Keywords:** Congenital abnormalities, intermittent positive-pressure ventilation, mediastinal diseases, paediatric emergency medicine

## Abstract

Mediastinal venolymphatic malformations (VLM) are rare tumours, with very few reported cases in the literature. Arising often from the anterior mediastinum, VLM manifests symptoms based on invaded surrounding structures. Masses from the anterior and superior mediastinum pose an anaesthetic challenge for airway and hemodynamic management. A 7-month-old male child presented with a progressively growing mass over the left anterior chest wall for one month, about 4x4 cm, with diffuse margins and now expanded to involve the root of the neck and into the axilla. The patient was free from any apparent systemic illness. The breathing difficulty worsened in the past week with noisy respiration associated with feeding difficulty and hence sought medical admission to the paediatrics emergency unit. In conclusion, such huge mediastinal masses are managed better under spontaneous ventilation with an adequate surgical depth of anaesthesia to maintain appropriate respiratory compliance and necessitate lower peak inspiratory pressure. Given rare cases reported in the literature, similar topics would help choose the modus of ventilation and their safe management.

**Main Points**

• Mediastinal VLMs are rare tumors with no clear-cut guidelines as to how to proceed.

• Moreover, whether to maintain the patient on spontaneous ventilation or perform surgery under muscle relaxation needs to be judged on a case-to-case basis and depends upon the expertise and logistics of the setup.

• Spontaneous ventilation is preferred in patients with difficult airways and with airway compromise.

## Introduction

Mediastinal venolymphatic malformations (VLM) are rare tumours, with very few reported cases in the literature. Arising often from the anterior mediastinum, VLM manifests symptoms based on invaded surrounding structures. Masses from the anterior and superior mediastinum pose an anaesthetic challenge for airway and hemodynamic management.^1^ The mass can compromise the airway, where the patient’s spontaneous ventilation and respiratory muscle tone may be indispensable in keeping the airway patent. Preservation of spontaneous ventilation and avoiding neuromuscular paralysis are desired to keep the airway patent by a trans-pleural gradient. Although not commonplace, cardiovascular compromise by anterior mediastinal masses can present with pericardial invasion, may compress the pulmonary artery or its branches, or cause superior vena cava compression.^2^ This can trigger a cardiovascular collapse on induction of general anaesthesia. A delicate balance of anaesthetic drug usage, ventilatory strategy and understanding of the mass effect will preempt any cardiopulmonary embarrassment.

## Case Presentation

**Figure 1:**
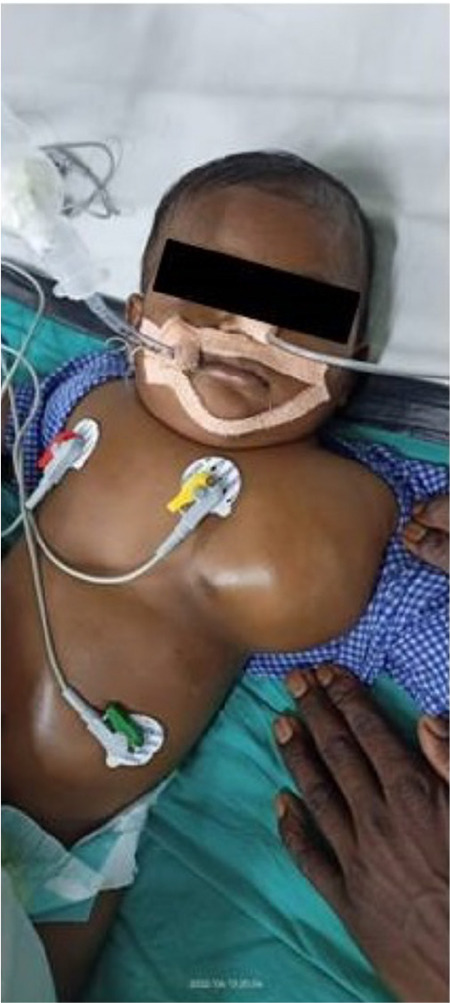
4 x 4 cm mass in the anterolateral part of the neck.

A 7-month-old male child weighing 9 kg presented with a progressively growing mass over the left anterior chest wall for one month, initially about 4 x 4 cm with diffuse margins and now expanded to involve the root of the neck and into the axilla (Figure 1). The patient was free from any apparent systemic illness. The breathing difficulty worsened in the past week with noisy respiration associated with feeding difficulty.

**Figure 2:**
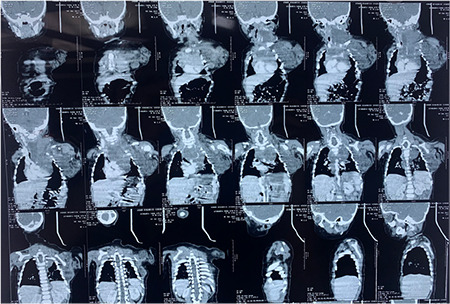
In the anterior, middle, and posterior mediastinum solid lesion measuring 10 cm (CC) x 7.5 cm (TR) x 7 cm (AP) and located primarily on the left side displacing the trachea, brachiocephalic artery, and superior vena cava towards the left side, encasing the arch of the aorta & its proximal branches and partially compressing the trachea above the diaphragm. Solid cystic lesion extending into the left axilla and along the left chest wall, measuring around 7.1 cm (TR) x 7.5 cm (AP) x 8 cm (CC).

A contrast-enhanced computerized tomography scan (Figure 2) of the chest showed a large heterogeneously hypodense mass lesion predominantly involving the upper and partially anterior, middle, and posterior mediastinum. Superiorly the lesion was cystic. In the neck, the lesion displaced left carotid vessels, completely compressing the left internal jugular vein, with mild compression of the right internal jugular vein. The larynx was displaced anteriorly and towards the right side.

**Figure 3:**
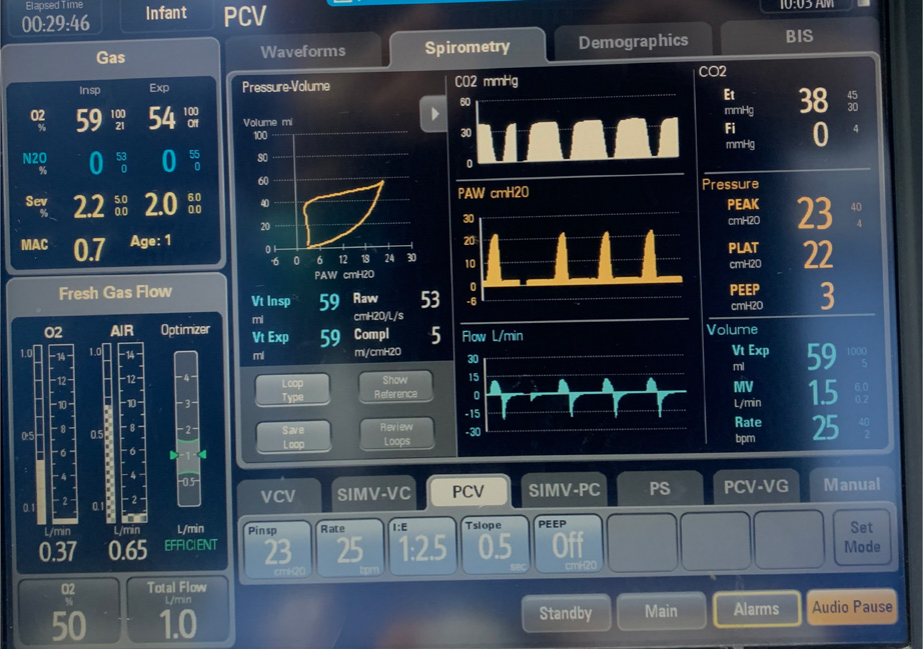
The monitor shows intra-operative ventilatory parameters as well as the compliance of the lungs.

The child presenting to the paediatric intensive care unit was dehydrated; the respiratory rate was 40/min^-1^, heart rate was 130 min^-1^, and SpO**_2_** was 96% on a face mask with 4 L of oxygen. There was biphasic audible stridor, indrawing of the chest wall, and intercostal retractions. The child was posted for surgery with informed consent from the parent. The patient was pre-oxygenated and sedated using intravenous (IV) midazolam, a bolus of 0.5 mg, and IV fentanyl with a bolus dose of 10 mcg for induction anaesthesia. The trachea was intubated using an uncuffed 4.0 ID endotracheal tube (ETT) and fixed at 12 cm. The child was ventilated on SIMV-PC mode with FiO_2_ of 0.4%, PEEP 6 hPa, and Pinsp of 16 hPa. On the day of surgery, the American Society of Anesthesiologist’s standard monitoring, anaesthesia gas monitoring, and invasive blood pressure was applied in the operating room. The tube position was confirmed with bilateral air entry on auscultation, Rocuronium 5 mg IV was administered, and the ventilatory mode was put to PCV with a Pinsp of 23 cmH_2_O (Figure 3). The tidal volume achieved was 50-60 mL, and end-tidal carbon dioxide was maintained between 35-40 mmHg. The dynamic compliance reflected was around 4-5 mL cmH_2_O and airway resistance of 53 cmH_2_O L^-1^ sec^-1^.

The surgery commenced, and the ventilation parameters remained the same after the sternotomy and the radiological findings were confirmed. After the muscle relaxation effects wore off, the patient was put on pressure support (PS) of 8 cmH_2_O, generating a tidal volume of 60-65 mL with a rate of 22-24 min^-1^. A multimodal analgesia plan comprising IV paracetamol, fentanyl, and ultrasound-guided bilateral erector spinae plane block at T3 & T5 levels with 0.125% of 14 mL ropivacaine and (1 mg) dexamethasone was instituted. The mass debulking involved extensive harmonic scalpel as vital neurovascular structures were in the vicinity or within the mas. The surgery subsequently had an uneventful course, and the child was shifted to the paediatric intensive care unit on ETT with CPAP support as the muscle relaxant effects wore off. The patient was extubated the next day in the afternoon and was on supplemental oxygen till the 5^th^ postoperative day. The patient was on a semisolid diet for the first two days following extubation and thereafter put on a solid diet. The mass had decreased by the 4^th^ postoperative day because of the bleomycin injection into the mass, and the rest of the course in the hospital was uneventful.

## Discussion

The cyclical pressure changes inside the thorax play a central role in regulating the heart and lung function.^1^ With mechanical ventilation, the atrial filling and cardiac output decrease due to increased intrathoracic pressure.^2^ This primarily affects the right ventricle (RV). The work done by the RV (afterload) mainly depends on pulmonary vascular resistance (PVR), which is affected by lung volumes.^2,3^ It is uncommon to see a clinically significant change in the PVR at physiological lung volumes and a PEEP of less than 10 cm H_2_O.^2^ However, mediastinal masses and chest wall tumours can undeniably reduce lung and chest wall compliance, hampering ventilation and compromising venous return. Spontaneous ventilation and avoiding muscle relaxant are advocated to reduce the risk of central airway occlusion in patients with mediastinal masses and even video-assisted thoracic surgeries.^4,5,6^ Problems with ventilation are often encountered after paralysis despite the apparent prior ability to ventilate. Hence short-acting paralytic agents, if needed, are preferred,^4,7,8^ usher the practice of spontaneous breathing being safer and better at preserving airway patency than positive pressure ventilation and paralysis.^5^ In a recent prospective observational study on seventeen adult patients with mediastinal mass, continuous video bronchoscopy recordings of the compromised airway portion were assessed in spontaneous and controlled ventilation modes. Hartigan et al.^5^ hypothesised that in adult patients with moderate to severe mediastinal mass-mediated tracheobronchial compression, anaesthetic interventions, including positive pressure ventilation and neuromuscular blockade, could be instituted without compromising central airway patency and challenged the general physiologic concepts regarding positive pressure ventilation and use of neuromuscular blocking agents.^5^ However, such an observation does not extend to paediatric airways since poor reserves and higher metabolic demand are present owing to a smaller field.^9^ Therefore, even though muscle relaxation was administered to ensure a still child, it increased the airway resistance as the splinting pressures were taken off and the respiratory dynamics were better maintained later with the PS mode of ventilation under deeper levels of sedation, as observed in our case. There are no clear-cut guidelines in such massive anterior mediastinal mass as to the mode of ventilation (spontaneous vs paralysis) and maintenance of the ventilation & oxygenation. The decision would depend on the morbidity of the patient and the involvement of the vital structures in the neck and anterior mediastinum, as conflicting views are found in the limited literature published about such a case.

To conclude, such huge mediastinal masses are better managed under spontaneous ventilation with an adequate surgical depth of anaesthesia to maintain appropriate respiratory compliance and necessitate lower peak inspiratory pressure. Such masses tend to involve the neurovascular structures in proximity, and their identification is paramount during debulking of the tumour. Similar rare cases in literature will help shed some insight into the ventilatory issues faced by this rare morbid condition.
